# Immunopathological Basis of Virus-induced Myocarditis

**DOI:** 10.1080/10446670410001670427

**Published:** 2004-03

**Authors:** Reinhard Maier, Philippe Krebs, Burkhard Ludewig

**Affiliations:** Research Department Kantonal Hospital St. Gallen St. Gallen 9007 Swaziland

## Abstract

Heart diseases are an important cause of morbidity and mortality in industrialized countries. Dilated cardiomyopathy (DCM), one of the most common heart diseases, may be the consequence of infectionassociated myocardits. Coxsackievirus B3 (CVB3) can be frequently detected in the inflamed heart muscle. CVB3-induced acute myocarditis is most likely the consequence of direct virus-induced myocyte damage, whereas chronic CVB3 infection-associated heart disease is dominated by its immunopathological sequelae. *Bona fide* autoimmunity, for example, directed against cardiac myosin, may favor chronic destructive immune damage in the heart muscle and thereby promote the development of DCM. The immunopathogenesis of myocarditis and subsequent DCM induced either by pathogens or autoantigens can be investigated in well-established animal models. In this article, we review recent studies on the role of viruses, with particular emphasis on CVB3, and different immunological effector mechanisms in initiation and progression of myocarditis.


*Clinical & Developmental Immunology*, March 2004, Vol. 11 (1), pp. 1–5

Taylor & Francis healthsciences

### Immunopathological Basis of Virus-induced Myocarditis

REINHARD MAIER, PHILIPPE KREBS and BURKHARD LUDEWIG*

*Research Department, Kantonal Hospital St. Gallen, 9007 St. Gallen, Switzerland*

Heart diseases are an important cause of morbidity and mortality in industrialized countries. Dilated cardiomyopathy (DCM), one of the most common heart diseases, may be the consequence of infection-associated myocardits. Coxsackievirus B3 (CVB3) can be frequently detected in the inflamed heart muscle. CVB3-induced acute myocarditis is most likely the consequence of direct virus-induced myocyte damage, whereas chronic CVB3 infection-associated heart disease is dominated by its immunopathological sequelae. *Bona fide* autoimmunity, for example, directed against cardiac myosin, may favor chronic destructive immune damage in the heart muscle and thereby promote the development of DCM. The immunopathogenesis of myocarditis and subsequent DCM induced either by pathogens or autoantigens can be investigated in well-established animal models. In this article, we review recent studies on the role of viruses, with particular emphasis on CVB3, and different immunological effector mechanisms in initiation and progression of myocarditis.

*Keywords*: Myocarditis; Dilated cardiomyopathy; Immunopathology; Bystander activation; Molecular mimicry

*Abbreviations*: CVB3, Coxsackievirus B3; CMV, cytomegalovirus; DCM, dilated cardiomyopathy

### INTRODUCTION

Infections with viruses, protozoan intracellular parasites or bacteria may be associated with myocarditis (Brown and O'Connell, 1995; Bowles *et al.*, 2003). Even vaccination efforts, such as recent vaccinia virus programs are a potential cause of cardiac adverse events (Murphy *et al.*, 2003). The most commonly known viral infectant of the heart are the Coxsackie B group viruses (CVB) in the Picornaviridae family. CVB3 RNA, for example, can be detected in the heart muscle of 40–50% of patients with dilated cardiomyopathy (DCM) (Fenoglio *et al.*, 1983; James, 1983; Dec *et al.*, 1985; Keating and Sanguinetti, 1996) and serological studies have shown a strong correlation between the presence of CVB3-specific antibodies and myocarditis (El-Hagrassy *et al.*, 1980). Furthermore, CVB3 infection has been proposed to be the major cause of pediatric myocarditis (Kaplan *et al.*, 1983).

Humans are the natural host for CVB3. The ability of this virus to infect mice and the susceptibility of specific mouse strains for CVB3-induced myocarditis provides a model system for the investigation of CVB3-induced immunopathological heart disease. Depending on the genetic background, the experimental disease in mice can be monophasic (acute myocarditis) or biphasic (acute myocarditis and subsequent low-grade inflammation). In the second phase of the biphasic myocarditis, infectious virus is usually no longer detectable and ongoing inflammation leads to heart muscle fibrosis and ventricular dilation. Mouse strains showing a biphasic disease progression are thus well-suited to study the shift from a viral infection with local tissue destruction to the induction of chronic immune responses to autoantigens (Kawai, 1999; Feldman *et al.*, 2000; Cunningham, 2001).

### ACUTE CVB3-INDUCED MYOCARDITIS

*In situ* hybridization and gene amplification studies support the notion that viral infection and persistence of viral RNA may lead to direct cytopathic damage of cardiomyocytes (Kandolf *et al.*, 1993; Klingel and Kandolf, 1993; Saraste *et al.*, 2003). The high cytopathogenic potential of CVB3 can be demonstrated *in vitro* by rapid lysis of cardiomyocytes (Herzum *et al.*, 1994), and *in vivo* in SCID mice lacking B- and T-cells, where the uncontrolled replication of CVB3 leads to severely enhanced myocardial damage (Chow *et al.*, 1992). Cell death occurring in the early phase of the infection may be a trigger for the subsequent inflammation. The early infiltrate in hearts of CVB3 infected mice consists of macrophages and T cells and a large proportion

*Corresponding author. Tel.: +41-71-494-1090. Fax: +41-71-494-6321. E-mail: burkhard.ludewig@kssg.ch

ISSN 1740-2522 print/ISSN 1740-2530 online © 2004 Taylor & Francis Ltd
DOI: 10.1080/10446670410001670427

of the T cells express the γδ T cell receptor (TCR) (Godeny and Gauntt, 1987a; Henke *et al.*, 1995). In CVB infected humans, macrophages and T cells are found among the infiltrating cells and a predominance of the Vβ7 TCR was described (Luppi *et al.*, 2003).

An early and efficient immune response is essential for the protection against cytopathic viruses (Zinkernagel, 1996). However, the immune response against CVB3 may be a double-edged sword. For example, depletion of αβT cells from CVB3-infected mice leads to a marked reduction in myocardial damage, despite comparable virus titers to control animals (Kishimoto and Abelmann, 1989). Likewise, γδT cells may contribute to immunopathological damage of cardiomyocytes (Huber, 2000; Huber *et al.*, 2001). A recent study by Huber and colleagues shows that CD1d-restricted γδT cells significantly promote the initial myocardial damage, but are not essential for viral clearance (Huber *et al.*, 2003). These results emphasize that it is most likely the quality of the initial anti-CVB3 response that sets the stage for the chronic immunopathological damage of the heart muscle.

### Innate Immune Responses in Early Virus-induced Myocarditis

Natural killer (NK) cells are able to inhibit the replication of CVB3 as shown by the fact that NK cell depletion leads to increased viral titers and more severe myocarditis (Godeny and Gauntt, 1987b; Godeny and Gauntt, 1986). The complement system may play a role in CVB3-induced myocarditis because C3, for example, is involved in antigen retention in germinal centers and viral clearance from spleen (Anderson *et al.*, 1997). Furthermore, it has been clearly shown for myosin-induced experimental autoimmune myocarditis that the presence of complement receptor type I and II expressed on a subset of T cells is important for myocarditis induction (Kaya *et al.*, 2001). Toll-like receptors (TLRs) are important for recognition of conserved microbial structures and subsequent activation of innate immune responses. In CVB3 infection, both myocarditis and viral replication are significantly reduced in the absence of TLR4 (Fairweather *et al.*, 2003). The availability of knockout mice for the several TLR types will help to further clarify the role of the innate immune system for CVB3-induced myocarditis.

### Adaptive Immune Responses in Virus-induced Myocarditis

The prominent role of T cells for the induction and/or severity of myocarditis is known since the pioneering work of Woodruff and Woodruff (1974) who showed that T cell-depleted mice suffer less from severe CVB-mediated myocarditis than control mice. Similarly, athymic nude mice show reduced disease after CVB3 infection (Hashimoto and Komatsu, 1978). Gene-deficient mice have been used to further delineate the immunopathological mechanisms in CVB3-induced myocarditis (Table I). CD8-deficient mice showed reduced survival after CVB3 infection, but showed no difference in the degree of myocarditis indicating that the contribution of CD8 T cells in immune-mediated heart inflammation is limited (Opavsky *et al.*, 1999). Studies in mice lacking the dominant effector molecule of cytotoxic T cells, perforin, provided conflicting results concerning the contribution of this molecule in CVB3 infection. Gebhard and colleagues showed that perforin-knockout mice survive CVB3 infection and that infection-associated myocarditis is less severe (Gebhard *et al.*, 1998). However, other studies could not detect significant differences between wild-type and perforin-knockout mice after CVB3 infection (Klingel *et al.*, 2003). The reason for these differences remains so far elusive, but the results from both studies clearly indicate that perforin is not essential for CVB3 elimination. An important role for T helper cells in the development of myocarditis is indicated by the fact that both CD4/CD8- and αβTCR-deficient mice show an increased survival rate and reduced myocardial damage after CVB3 infection (Opavsky *et al.*, 1999). Furthermore, CVB3 elicits only mild myocarditis in mice lacking only the CD4 molecule (Opavsky *et al.*, 1999). Importantly, in this series of experiments, virus titers in the hearts of CD8-, CD4-, C4/CD8- and TCR-αβ-deficient mice were comparable in the first week post infection (Opavsky *et al.*, 1999) demonstrating that the extent of myocardial injury is mainly determined by immunopathological mechanisms.

TABLE I Impact of gene deficiencies on Coxsackievirus B3-induced myocarditis

| Knock-out | Effect on CVB myocarditis | Effect on survival | Strain | Reference |
|---|---|---|---|---|
| CD8 | None | Reduced | A/J | Opavsky *et al.* (1999) |
| CD4 | Reduced | None | A/J | Opavsky *et al.* (1999) |
| CD4/CD8 | Reduced | Reduced | A/J | Opavsky *et al.* (1999) |
| TCR α/β | Reduced | Reduced | A/J | Opavsky *et al.* (1999) |
| β2-microglobulin | Increased | None | C57BL/6 | Klingel *et al.* (2003) |
| Perforin | None | None | C57BL/6 | Klingel *et al.* (2003) |
| Perforin | Reduced | Increased | C57BL/6 | Gebhard *et al.* (1998) |
| TCR-Jα281 | None | None | Balb/c | Huber *et al.* (2003) |
| CD1d | Reduced | Increased | Balb/c | Huber *et al.* (2003) |

Recent work supports the notions that γδ T cells play a prominent role in CVB3-induced myocarditis (Huber, 2000; Huber *et al.*, 2002). The finding that at least a fraction of these γδ T cells are CD1d-restricted (Huber *et al.*, 2003) will stimulate the search for unconventional (lipid?) antigens that trigger γδ TCRs in myocarditis.

Infection of humans and mice by CVB3 as well as MCMV infection of mice induces the production of autoantibodies to cardiac myosin and other heart antigens (Neu *et al.*, 1990; O'Donoghue *et al.*, 1990; Lauer *et al.*, 1994). These antibodies appear earliest on day 7 post infection in CVB3 infected mice and can be eluted from the heart muscle (Neumann *et al.*, 1994; Latif *et al.*, 1999). The question that arises is whether these autoantibodies play a critical role for the onset and progression of autoimmune myocarditis. Studies on CVB3 infection in B cell-deficient mice indicated that antibodies are not important for the development of myocarditis and disease progression, but play a prominent role in the control of the infection (Mena *et al.*, 1999). However, B cells secreting heart-specific autoantibodies may impact on immune-mediated myocardial inflammation. For example, anti-myosin antibodies can mediate myocarditis in susceptible mouse strains and disease susceptibility depends on the presence of myosin or a myosin-like molecule in cardiac extracellular matrix (Liao *et al.*, 1995). Furthermore, a recent report revealed that DCM in the negative immune regulatory receptor PD-1-knockout mice is mediated by autoantibodies reactive to cardiomyocytes (Nishimura *et al.*, 2001). Autoantibodies recognizing extracellularly deposited cardiac troponin I (cTn I) induced dilatation and dysfunction of the heart by induction of a permanent influx of $Ca^{2+}$ in cardiomyocytes. Interestingly, the presence of cTn I-specific antibodies in the heart did not induce inflammation, despite the formation of immune complexes (Okazaki *et al.*, 2003). It is noteworthy that in this particular experimental setting, myocarditis is not a prerequisite for the development of immune-mediated DCM.

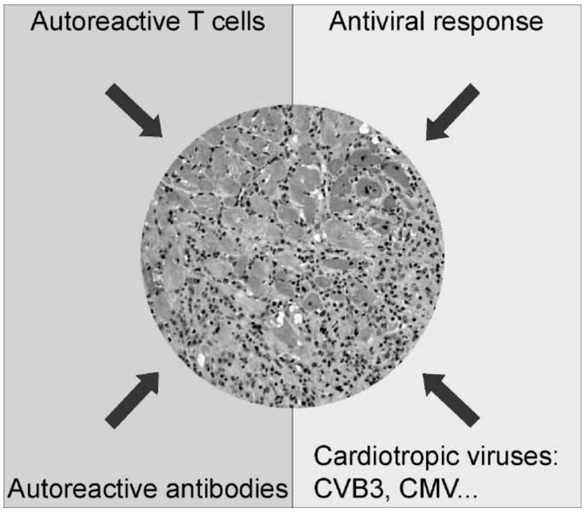

FIGURE 1 Immunopathogenesis of myocarditis. The inlet shows massive mononuclear cell infiltration and cardiomyocyte necrosis in a heart section of a myosin peptide-immunized Balb/c mouse (HE stain, magnification: × 100).

### Immune Mechanisms in Autoimmune Myocarditis

The best-studied cardiac autoantigen is the alpha chain of cardiac myosin. Immunization of susceptible mice with myosin protein or defined, MHC class II restricted myosin peptides induces myocarditis (Neu *et al.*, 1987) (see Fig. 1). This experimental approach uncouples the chronic phase from the virus infection, and is therefore a valuable tool to study the mechanisms and effector molecules involved in autoimmune myocarditis and to evaluate therapeutic strategies. As shown in adoptive transfer experiments, the inflammatory response in autoimmune myocarditis is mediated by Th cells. Transfer of CD4 T cells from peptide-immunized mice induces myocarditis in non-immunized mice (Smith and Allen, 1991). In addition, the injection of anti-MHC II antibodies prevents disease (Pummerer *et al.*, 1991). Induction of autoimmune myocarditis in mice strongly depends on the genetic background and the MHC II locus (Neu *et al.*, 1987). Likewise, human chronic heart disease has also been correlated to certain HLA alleles and experiments in transgenic mice expressing human CD4 and HLA-DQ6 show that genetically resistant C57BL/6 mice may become susceptible to autoimmune myocarditis (Bachmaier *et al.*, 1999). Autoimmune damage in the heart by Th cells is most likely mediated via local interaction with professional antigen-presenting cells (APCs) because myocytes do not express MHC II molecules. It has been demonstrated that expression of myosin-MHC II complexes are presented by resident heart APC and that the presentation of this antigen precedes the infiltration of T cells (Smith and Allen, 1992). Furthermore, MHC II upregulation in the heart is a prerequisite for disease induction as shown in tumor necrosis factor (TNF)-Rp55-deficient mice, where MHC II upregulation is abrogated (Bachmaier *et al.*, 1997). Other proinflammotory cytokines such as IL-12 and IFN-γ have been shown to be involved in the pathogenesis of autoimmune myocarditis. IL-12, like TNF-α, is important for the onset of disease and IFN-γ seems to play a protective role via the induction of nitric oxide (Afanasyeva *et al.*, 2001; Eriksson *et al.*, 2001a,b). As mentioned above, complement receptors I and II expression on a subset of T cells is important for the onset of autoimmune myocarditis (Kaya *et al.*, 2001). Furthermore, it was shown that dendritic cell-induced autoimmune myocarditis requires cooperation between adaptive and innate immune responses (Eriksson *et al.*, 2003). Further studies using transgenic mice and genetically modified viruses will help to shed light on the complex mechanisms operative in auto-immune myocarditis.

### CONCLUSION

The etiological link between infection, myocarditis and subsequent DCM is well-established in humans. Furthermore, the shift from pathogen-induced myocarditis to an autoimmune-mediated chronic degenerative heart disease is well-documented in animal models. It has been proposed that breaking of self-tolerance to autoantigens is mediated by molecular mimicry, bystander activation or epitope spreading (Vanderlugt and Miller, 2002). Although the contribution of these immunopathological mechanisms has been studied in other virus infections such as Theiler's virus (Vanderlugt and Miller, 2002) or lymphocytic choriomeningitis virus (Zinkernagel, 2002), a detailed analysis for CVB3 infection is still lacking. The knowledge how CVB3 and maybe other infectious agents cause myocarditis and subsequent DCM will make these diseases amenable to new treatment options such as immunosuppression (Frustaci *et al.*, 2002) or immunomodulation (Fig. 1).

### *Acknowledgements*

This work was supported by the Swiss National Science Foundation and the Kanton of St. Gallen.

### *References*

Afanasyeva, M., Wang, Y., Kaya, Z., *et al.* (2001) "Interleukin-12 receptor/STAT4 signaling is required for the development of autoimmune myocarditis in mice by an interferon-gamma-independent pathway", *Circulation* **104**, 3145–3151.

Anderson, D.R., Carthy, C.M., Wilson, J.E., Yang, D., Devine, D.V. and McManus, B.M. (1997) "Complement component 3 interactions with coxsackievirus B3 capsid proteins: innate immunity and the rapid formation of splenic antiviral germinal centers", *J. Virol.* **71**, 8841–8845.

Bachmaier, K., Pummerer, C., Kozieradzki, I., *et al.* (1997) "Low-molecular-weight tumor necrosis factor receptor p55 controls induction of autoimmune heart disease", *Circulation* **95**, 655–661.

Bachmaier, K., Neu, N., Yeung, R.S., Mak, T.W., Liu, P. and Penninger, J.M. (1999) "Generation of humanized mice susceptible to peptide-induced inflammatory heart disease", *Circulation* **99**, 1885–1891.

Bowles, N.E., Vallejo, J., Javier Fuentes-Garcia, F., *et al.* (2003) "Viral causes of cardiac inflammation", *Curr. Opin. Cardiol.* **18**, 182–188.

Brown, C.A. and O'Connell, J.B. (1995) "Myocarditis and idiopathic dilated cardiomyopathy", *Am. J. Med.* **99**, 309–314.

Chow, L.H., Beisel, K.W. and McManus, B.M. (1992) "Enteroviral infection of mice with severe combined immunodeficiency. Evidence for direct viral pathogenesis of myocardial injury", *Lab. Investig.* **66**, 24–31.

Cunningham, M.W. (2001) "Cardiac myosin and the TH1/TH2 paradigm in autoimmune myocarditis", *Am. J. Pathol.* **159**, 5–12.

Dec, Jr., G.W., Palacios, I.F., Fallon, J.T., *et al.* (1985) "Active myocarditis in the spectrum of acute dilated cardiomyopathies. Clinical features, histologic correlates, and clinical outcome", *N. Engl. J. Med.* **312**, 885–890.

El-Hagrassy, M.M., Banatvala, J.E. and Coltart, D.J. (1980) "Coxsackie-B-virus-specific IgM responses in patients with cardiac and other diseases", *Lancet* **2**, 1160–1162.

Eriksson, U., Kurrer, M.O., Sebald, W., Brombacher, F. and Kopf, M. (2001) "Dual role of the IL-12/IFN-gamma axis in the development of autoimmune myocarditis: induction by IL-12 and protection by IFN-gamma", *J. Immunol.* **167**, 5464–5469.

Eriksson, U., Kurrer, M.O., Bingisser, R., *et al.* (2001) "Lethal autoimmune myocarditis in interferon-gamma receptor-deficient mice: enhanced disease severity by impaired inducible nitric oxide synthase induction", *Circulation* **103**, 18–21.

Eriksson, U., Ricci, R., Hunziker, L., *et al.* (2003) "Dendritic cell-induced autoimmune heart failure requires cooperation between adaptive and innate immunity", *Nat. Med.* **9**, 1484–1490.

Fairweather, D., Yusung, S., Frisancho, S., *et al.* (2003) "IL-12 receptor beta 1 and Toll-like receptor 4 increase IL-1 beta- and IL-18-associated myocarditis and coxsackievirus replication", *J. Immunol.* **170**, 4731–4737.

Feldman, A.M. and McNamara, D. (2000) "Myocarditis", *N. Engl. J. Med.* **343**, 1388–1398.

Fenoglio, Jr., J.J., Ursell, P.C., Kellogg, C.F., Drusin, R.E. and Weiss, M.B. (1983) "Diagnosis and classification of myocarditis by endomyocardial biopsy", *N. Engl. J. Med.* **308**, 12–18.

Frustaci, A., Cuoco, L., Chimenti, C., *et al.* (2002) "Celiac disease associated with autoimmune myocarditis", *Circulation* **105**, 2611–2618.

Gebhard, J.R., Perry, C.M., Harkins, S., *et al.* (1998) "Coxsackievirus B3-induced myocarditis: perforin exacerbates disease, but plays no detectable role in virus clearance", *Am. J. Pathol.* **153**, 417–428.

Godeny, E.K. and Gauntt, C.J. (1986) "Involvement of natural killer cells in coxsackievirus B3-induced murine myocarditis", *J. Immunol.* **137**, 1695–1702.

Godeny, E.K. and Gauntt, C.J. (1987) "*In situ* immune autoradiographic identification of cells in heart tissues of mice with coxsackievirus B3-induced myocarditis", *Am. J. Pathol.* **129**, 267–276.

Godeny, E.K. and Gauntt, C.J. (1987) "Murine natural killer cells limit coxsackievirus B3 replication", *J. Immunol.* **139**, 913–918.

Hashimoto, I. and Komatsu, T. (1978) "Myocardial changes after infection with Coxsackie virus B3 in nude mice", *Br. J. Exp. Pathol.* **59**, 13–20.

Henke, A., Huber, S., Stelzner, A. and Whitton, J.L. (1995) "The role of CD8 + T lymphocytes in coxsackievirus B3-induced myocarditis", *J. Virol.* **69**, 6720–6728.

Herzum, M., Ruppert, V., Kuytz, B., Jomaa, H., Nakamura, I. and Maisch, B. (1994) "Coxsackievirus B3 infection leads to cell death of cardiac myocytes", *J. Mol. Cell Cardiol.* **26**, 907–913.

Huber, S.A. (2000) "T cells expressing the gamma delta T cell receptor induce apoptosis in cardiac myocytes", *Cardiovasc. Res.* **45**, 579–587.

Huber, S.A., Graveline, D., Born, W.K., *et al.* (2001) "Cytokine production by Vgamma(+)-T-cell subsets is an important factor determining CD4(+)-Th-cell phenotype and susceptibility of BALB/c mice to coxsackievirus B3-induced myocarditis", *J. Virol.* **75**, 5860–5869.

Huber, S., Shi, C. and Budd, R.C. (2002) "Gammadelta T cells promote a Th1 response during coxsackievirus B3 infection *in vivo*: role of Fas and Fas ligand", *J. Virol.* **76**, 6487–6494.

Huber, S., Sartini, D. and Exley, M. (2003) "Role of CD1d in coxsackievirus B3-induced myocarditis", *J. Immunol.* **170**, 3147–3153.

James, T.N. (1983) "Myocarditis and cardiomyopathy", *N. Engl. J. Med.* **308**, 39–41.

Kandolf, R., Klingel, K., Zell, R., *et al.* (1993) "Molecular mechanisms in the pathogenesis of enteroviral heart disease: acute and persistent infections", *Clin. Immunol. Immunopathol.* **68**, 153–158.

Kaplan, M.H., Klein, S.W., McPhee, J. and Harper, R.G. (1983) "Group B coxsackievirus infections in infants younger than three months of age: a serious childhood illness", *Rev. Infect. Dis.* **5**, 1019–1032.

Kawai, C. (1999) "From myocarditis to cardiomyopathy: mechanisms of inflammation and cell death: learning from the past for the future", *Circulation* **99**, 1091–1100.

Kaya, Z., Afanasyeva, M., Wang, Y., *et al.* (2001) "Contribution of the innate immune system to autoimmune myocarditis: a role for complement", *Nat. Immunol.* **2**, 739–745.

Keating, M.T. and Sanguinetti, M.C. (1996) "Molecular genetic insights into cardiovascular disease", *Science* **272**, 681–685.

Kishimoto, C. and Abelmann, W.H. (1989) "Monoclonal antibody therapy for prevention of acute coxsackievirus B3 myocarditis in mice", *Circulation* **79**, 1300–1308.

Klingel, K. and Kandolf, R. (1993) "The role of enterovirus replication in the development of acute and chronic heart muscle disease in different immunocompetent mouse strains", *Scand J. Infect. Dis. Suppl.* **88**, 79–85.

Klingel, K., Schnorr, J.J., Sauter, M., Szalay, G. and Kandolf, R. (2003) "beta2-Microglobulin-associated regulation of interferon-gamma and virus-specific immunoglobulin G confer resistance against the development of chronic coxsackievirus myocarditis", *Am. J. Pathol.* **162**, 1709–1720.

Latif, N., Zhang, H., Archard, L.C., Yacoub, M.H. and Dunn, M.J. (1999) "Characterization of anti-heart antibodies in mice after infection with coxsackie B3 virus", *Clin. Immunol.* **91**, 90–98.

Lauer, B., Padberg, K., Schultheiss, H.P. and Strauer, B.E. (1994) "Autoantibodies against human ventricular myosin in sera of patients with acute and chronic myocarditis", *J. Am. Coll. Cardiol.* **23**, 146–153.

Liao, L., Sindhwani, R., Rojkind, M., Factor, S., Leinwand, L. and Diamond, B. (1995) "Antibody-mediated autoimmune myocarditis depends on genetically determined target organ sensitivity", *J. Exp. Med.* **181**, 1123–1131.

Luppi, P., Rudert, W., Licata, A., *et al.* (2003) "Expansion of specific alphabeta + T-cell subsets in the myocardium of patients with myocarditis and idiopathic dilated cardiomyopathy associated with Coxsackievirus B infection", *Hum. Immunol.* **64**, 194–210.

Mena, I., Perry, C.M., Harkins, S., Rodriguez, F., Gebhard, J. and Whitton, J.L. (1999) "The role of B lymphocytes in coxsackievirus B3 infection", *Am. J. Pathol.* **155**, 1205–1215.

Murphy, J.G., Wright, R.S., Bruce, G.K., *et al.* (2003) "Eosinophilic-lymphocytic myocarditis after smallpox vaccination", *Lancet* **362**, 1378–1380.

Neu, N., Rose, N.R., Beisel, K.W., Herskowitz, A., Gurri-Glass, G. and Craig, S.W. (1987) "Cardiac myosin induces myocarditis in genetically predisposed mice", *J. Immunol.* **139**, 3630–3636.

Neu, N., Ploier, B. and Ofner, C. (1990) "Cardiac myosin-induced myocarditis. Heart autoantibodies are not involved in the induction of the disease", *J. Immunol.* **145**, 4094–4100.

Neumann, D.A., Rose, N.R., Ansari, A.A. and Herskowitz, A. (1994) "Induction of multiple heart autoantibodies in mice with coxsackievirus B3- and cardiac myosin-induced autoimmune myocarditis", *J. Immunol.* **152**, 343–350.

Nishimura, H., Okazaki, T., Tanaka, Y., *et al.* (2001) "Autoimmune dilated cardiomyopathy in PD-1 receptor-deficient mice", *Science* **291**, 319–322.

O'Donoghue, H.L., Lawson, C.M. and Reed, W.D. (1990) "Autoantibodies to cardiac myosin in mouse cytomegalovirus myocarditis", *Immunology* **71**, 20–28.

Okazaki, T., Tanaka, Y., Nishio, R., *et al.* (2003) "Autoantibodies against cardiac troponin I are responsible for dilated cardiomyopathy in PD-1-deficient mice", *Nat. Med.* **9**, 1477–1483.

Opavsky, M.A., Penninger, J., Aitken, K., *et al.* (1999) "Susceptibility to myocarditis is dependent on the response of alphabeta T lymphocytes to coxsackieviral infection", *Circ. Res.* **85**, 551–558.

Pummerer, C., Berger, P., Fruhwirth, M., Ofner, C. and Neu, N. (1991) "Cellular infiltrate, major histocompatibility antigen expression and immunopathogenic mechanisms in cardiac myosin-induced myocarditis", *Lab. Investig.* **65**, 538–547.

Saraste, A., Arola, A., Vuorinen, T., *et al.* (2003) "Cardiomyocyte apoptosis in experimental coxsackievirus B3 myocarditis", *Cardiovasc. Pathol.* **12**, 255–262.

Smith, S.C. and Allen, P.M. (1991) "Myosin-induced acute myocarditis is a T cell-mediated disease", *J. Immunol.* **147**, 2141–2147.

Smith, S.C. and Allen, P.M. (1992) "Expression of myosin-class II major histocompatibility complexes in the normal myocardium occurs before induction of autoimmune myocarditis", *Proc. Natl. Acad. Sci. USA* **89**, 9131–9135.

Van der Lugt, C.L. and Miller, S.D. (2002) "Epitope spreading in immune-mediated diseases: implications for immunotherapy", *Nat. Rev. Immunol.* **2**, 85–95.

Woodruff, J.F. and Woodruff, J.J. (1974) "Involvement of T lymphocytes in the pathogenesis of coxsackie virus B3 heart disease", *J. Immunol.* **113**, 1726–1734.

Zinkernagel, R.M. (1996) "Immunology taught by viruses", *Science* **271**, 173–178.

Zinkernagel, R.M. (2002) "Antiinfection immunity and autoimmunity", *Ann. NY Acad. Sci.* **958**, 3–6.

